# Pharmacokinetics and excretion of ^14^C-omacetaxine in patients with advanced solid tumors

**DOI:** 10.1007/s10637-016-0360-9

**Published:** 2016-05-25

**Authors:** Cynthia M. Nijenhuis, Edward Hellriegel, Jos H. Beijnen, Diane Hershock, Alwin D. R. Huitema, Luc Lucas, Marja Mergui-Roelvink, Mihaela Munteanu, Laura Rabinovich-Guilatt, Philmore Robertson, Hilde Rosing, Ofer Spiegelstein, Jan H. M. Schellens

**Affiliations:** 1 Department of Pharmacy & Pharmacology, Antoni van Leeuwenhoek/The Netherlands Cancer Institute and MC Slotervaart, Amsterdam, The Netherlands; 2 Teva Branded Pharmaceutical Products R&D, Nonclinical DMPK, West Chester, PA USA; 3 Division of Pharmacoepidemiology and Clinical Pharmacology, Faculty of Science, Department of Pharmaceutical Sciences, Utrecht University, Utrecht, The Netherlands; 4 Teva Branded Pharmaceutical Products R&D, Oncology Clinical Development, Frazer, PA USA; 5 Division of Clinical Pharmacology, Department of Medical Oncology, The Netherlands Cancer Institute, Amsterdam, The Netherlands; 6 ImmunoGen, Inc, Waltham, MA USA; 7 Teva Global Branded Products, 41 Moores Road, PO Box 4011, Frazer, PA 19355 USA; 8 Teva Global Branded Products, Netanya, Israel

**Keywords:** Omacetaxine mepesuccinate, Pharmacokinetics, Excretion, Mass balance, Metabolism

## Abstract

*Background* Omacetaxine mepesuccinate is indicated in adults with chronic myeloid leukemia resistant and/or intolerant to ≥ 2 tyrosine kinase inhibitor treatments. This phase I study assessed the disposition, elimination, and safety of ^14^C-omacetaxine in patients with solid tumors. *Methods* The study comprised a 7-days pharmacokinetic assessment followed by a treatment period of ≤ six 28-days cycles. A single subcutaneous dose of 1.25 mg/m^2^^14^C-omacetaxine was administered to six patients. Blood, urine, and feces were collected through 168 h or until radioactivity excreted within 24 h was <1 % of the dose. Total radioactivity (TRA) was measured in all matrices and concentrations of omacetaxine, 4′-desmethylhomoharringtonine (4′-DMHHT), and cephalotaxine were measured in plasma and urine. For each treatment cycle, patients received 1.25 mg/m^2^ omacetaxine twice daily for 7 days. *Results* Mean TRA recovered was approximately 81 % of the dose, with approximately half of the radioactivity recovered in feces and half in urine. Approximately 20 % of the dose was excreted unchanged in urine; cephalotaxine (0.4 % of dose) and 4′ DMHHT (9 %) were also present. Plasma concentrations of TRA were higher than the sum of omacetaxine and known metabolites, suggesting the presence of other ^14^C-omacetaxine-derived compounds. Fatigue and anemia were common, consistent with the known toxicity profile of omacetaxine. *Conclusion* Renal and hepatic processes contribute to the elimination of ^14^C-omacetaxine-derived radioactivity in cancer patients. In addition to omacetaxine and its known metabolites, other ^14^C-omacetaxine-derived materials appear to be present in plasma and urine. Omacetaxine was adequately tolerated, with no new safety signals.

## Introduction

**Fig. 1:**
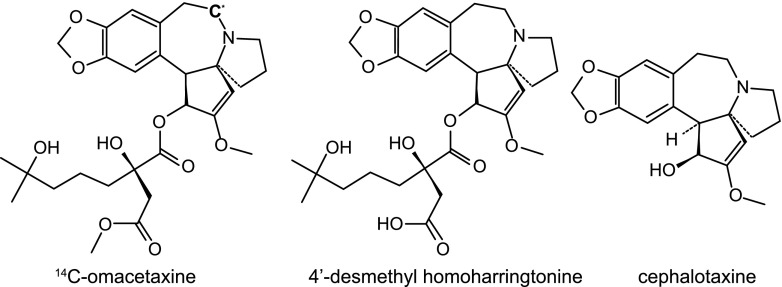
Chemical structure of ^14^C-omacetaxine and its known metabolites 4′-DMHHT and cephalotaxine. The asterisk in the ^14^C-omacetaxine structure indicates the position of the ^14^C-label

Omacetaxine mepesuccinate (henceforth referred to as omacetaxine, Fig. 1) is a cephalotaxine ester that is approved by the US Food and Drug Administration (FDA) as Synribo® for the treatment of adult patients with chronic myeloid leukemia (CML) with resistance and/or intolerance to two or more tyrosine kinase inhibitors [1]. Omacetaxine is a semisynthetic product from the leaves of *Cephalotaxus fortunei*; the chemical structure of omacetaxine is identical to that of the natural product homoharringtonine, found in the bark of this tree [2]. Omacetaxine is a protein synthesis inhibitor that has demonstrated activity in CML, acute promyelocytic leukemia, acute myelogenous leukemia, and myelodysplastic syndrome [1, 3–11]. Omacetaxine’s activity is independent of direct binding to breakpoint cluster region-abelson (Bcr-Abl) tyrosine kinase. Instead, it binds to the A-side cleft of ribosomes, thus reducing levels of multiple short-lived oncoproteins involved in cell survival and proliferation pathways [12, 13]. For patients with CML, the induction dose is 1.25 mg/m^2^ administered by subcutaneous injection twice daily for 14 days every 28 days; the maintenance dosage and route are the same as for induction, with omacetaxine administered for 7 days of a 28-days cycle [14].

To date, little is known about the metabolism, disposition, and elimination of omacetaxine. In a previous in vivo metabolite study in mice, 4′-desmethylhomoharringtonine (4′-DMHHT, Fig. 1) was the primary metabolite identified [15]. In mice, conversion occurred quickly (within 5 min of intravenous administration), and in vitro assessments suggested that the process of hydrolysis was mediated primarily by plasma esterase [15]. The formation of 4′-DMHHT was also shown to occur when omacetaxine was incubated with liver microsomes isolated from rats and rabbits [16]. Cephalotaxine (Fig. 1) is a minor, inactive metabolite of omacetaxine [15]. In a previous phase I study, 4′-DMHHT and cephalotaxine concentrations were determined in plasma and urine; cephalotaxine was undetectable in most patients, and the steady-state area under the curve (AUC) estimate for 4′-DMHHT was approximately 13 % of that for omacetaxine [17].

The primary objective of the present study was to characterize the disposition and elimination pathway of ^14^C-omacetaxine in patients with solid tumors or relapsed/refractory hematologic malignancies.

## Material and methods

### Study design

This was a phase I, open-label, single-institution study conducted in accordance with International Conference on Harmonisation guidelines for Good Clinical Practice, the US Code of Federal Regulations, and the European Union Directive. The protocol was approved by The Netherlands Cancer Institute Independent Ethics Committee. All patients provided written, informed consent at the time of screening.

The study was divided into two assessment periods; period A comprised 7 days during which the mass balance and pharmacokinetics of ^14^C-omacetaxine were investigated, and period B was an extended-use period of 28-days cycles with nonlabeled omacetaxine. Safety was assessed during both periods.

### Patients

Eligible patients were at least 18 years of age and had histologically or cytologically confirmed relapsed and/or refractory hematologic malignancies or advanced solid tumors considered unresponsive or poorly responsive to standard care. Only patients with advanced solid tumors participated; none had hematologic malignancies. Other eligibility criteria included World Health Organization performance status of 2 or lower; estimated life expectancy of at least 3 months; QTc less than 450 msec; normal hepatic function (defined as ≤ upper limit of the normal range [ULN] for aspartate aminotransferase [AST] and total bilirubin, or mild hepatic dysfunction (defined as bilirubin ≤ 1.5 times the ULN and AST greater than the ULN, or bilirubin > 1.0 to 1.5 times the ULN and any AST); and adequate renal function (creatinine clearance ≥ 60 mL/min). Patients with nonhematologic malignancies were required to have absolute neutrophil counts of at least 1000 cells/mm^3^, platelet counts of at least 100,000 cells/mm^3^, and hemoglobin values of at least 8 g/dL. Male patients were required to be surgically sterile or currently using an approved method of birth control for at least 90 days after drug discontinuation, and female patients were required to be surgically sterile or 2 years postmenopausal.

Patients were excluded if they had received mitomycin C within 42 days; chemotherapy, radiotherapy, radioimmunotherapy, or immunotherapy within 28 days; or hematopoietic growth factors within 14 days prior to the first dose or if they had not recovered from adverse events (AEs) caused by previously administered agents. Other reasons for exclusion included pregnancy or breastfeeding; New York Heart Association Class III or IV heart disease; myocardial infarction; solid tumor with symptomatic central nervous system metastases; systemic infection or medical/psychiatric condition; other treatments for hematologic or nonhematologic malignancy; known hypersensitivity to omacetaxine, mannitol, or other components of the study drug; previous treatment with omacetaxine; or significant constipation or obstruction of the urinary tract.

### Study treatment

Labeled ^14^C-omacetaxine (chemical purity 99 %, radiochemical purity 98 %, chiral purity 100 %) was prepared by Selcia Limited (Ongar, Essex, UK) and supplied by Teva Pharmaceuticals (North Wales, PA, USA). Drug product for period A was manufactured for each patient by PRA Early Development Services (Zuidlaren, The Netherlands) and contained a mixture of ^14^C-omacetaxine and nonlabeled omacetaxine in a 1-mL plastic syringe, with approximately 95 μCi of ^14^C. The specific activity and concentration of the mixture were approximately 135 μCi/mL and 3 mg/mL, respectively. Therefore, both the ^14^C-labeled and nonlabeled dose were adjusted based on the body surface area (BSA) of each individual patient. Nonlabeled omacetaxine was provided in a lyophilized vial containing 3.5 mg of omacetaxine and 10 mg of mannitol in an 8-mL clear glass vial by Teva Pharmaceuticals.

On day 1 of period A, each patient received a single subcutaneous dose of ^14^C-omacetaxine 1.25 mg/m^2^ at the study center. The first 72 h of assessment in period A were conducted on an inpatient basis. Blood samples and excreta were collected for up to 168 h after administration. If necessary, excreta collections were continued on an outpatient basis until total radioactivity (TRA) excreted in the urine and feces collections in a 24-h period were less than 1 % of the administered dose. The TRA measurements for each patient were adjusted based on the specific activity of the individual dose. During period A, patients received a high-fiber diet and adequate fluid intake (≥2 L/day) to expedite intestinal transit and reduce the potential for retention of radioactivity.

Period B started on day 4 of period A (following the 72-h pharmacokinetic sample collection). During period B, patients were treated with nonlabeled omacetaxine 1.25 mg/m^2^ subcutaneously twice daily for 7 days (solid tumors) or 14 days (hematologic malignancies) of each 28-days cycle for a maximum of six cycles. If this dose was not tolerated, it could be started at or lowered to 1.0 mg/m^2^, as allowed in the protocol, and the number of dosing days modified.

### Sample collection

For pharmacokinetics analyses, blood samples of 6 mL were collected in K_2_EDTA tubes prior to the injection and at 15, 30, 45, and 60 min and 2, 4, 8, 12, 24, 32, 48, and 72 h after ^14^C-omacetaxine injection, and 4-mL samples were collected 96, 120, 144, and 168 h after injection. The tubes were placed in ice water after collection and centrifuged (1800 g, 4 °C, 15 min) within 5 min. Plasma was isolated and aliquoted for TRA measurements; samples collected through 72 h were stabilized with 5 % of a 0.4 % ethanolic paraoxon solution for bioanalysis. Paraoxon has been shown to be an effective inhibitor of omacetaxine hydrolysis as mediated by plasma esterases [18]. In addition, 1 mL of whole blood was collected 30 min and 8, 72, and 168 h after injection and optionally twice every week thereafter for TRA measurements.

Urine samples were collected before ^14^C-omacetaxine injection and then as voided through 168 h after injection, until the total excreted radioactivity per day (urine and feces combined) was less than 1 % of the total administered dose (whichever was earlier) or through more than 168 h if TRA represented at least 1 % of the radiochemical dose in the 144- to 168-h collection period. Each urine sample was aliquoted for measurement of TRA, and additional aliquots for samples collected through 72 h were prepared for bioanalysis.

Fecal samples were collected per portion, before ^14^C-omacetaxine injection and then as voided through 168 h after injection, until the total excreted radioactivity per day was less than 1 % of the total administered dose (whichever was earlier), or longer if TRA represented at least 1 % of the radiochemical dose in the 144- to 168-h collection of feces. The fecal portions were weighed, stored refrigerated, and homogenized after addition of water (1:3 *w/v*). The homogenized fecal samples were aliquoted for measurement of TRA.

Plasma aliquots, urine aliquots, whole blood aliquots, and fecal homogenate aliquots were stored within a range between −70 and −90 °C. The plasma, urine, and fecal samples collected were also used for metabolic profiling and identification. The results of these analyses are reported elsewhere [18].

### Total radioactivity analysis

The TRA in plasma, whole blood, urine, and feces was determined by liquid scintillation counting. Plasma (0.2 mL) and urine (1 mL) samples were mixed directly with 10 mL liquid scintillation cocktail (Ultima Gold^TM^, Perkin Elmer Inc., Waltham, MA, USA). To the whole blood samples (0.2 mL), 1 mL Solvable (Perkin Elmer Inc.), 0.1 mL 0.1 M EDTA, and 0.5 mL 30 % hydrogen peroxide were added to dissolve and decolorize the samples. Feces homogenates (0.2 mL) were first dissolved and decolorized using 1 mL Solvable (Perkin Elmer Inc.), 1 mL isopropanol, and 0.4 mL 30 % hydrogen peroxide. The decolorization reaction was started by warming the samples in a shaking water bath of approximately 43 °C, after which the samples were placed in a dark cool place for at least 1 h before liquid scintillation cocktail (10 mL) was added. Samples were counted on a Tri-Carb® 2800TR liquid scintillation counter (Perkin Elmer Inc.). Quench correction was applied with a calibration curve of quenched radioactive reference standards. Samples were counted to a sigma 2 counting error of 5 % or for 60 min at most. The lower limit of quantitation (LLOQ) was approximated to be 1.2 to 1.4 ng-eqv/g using the counting error as described previously [18, 19].

### Analysis of omacetaxine, 4′-DMHHT, and cephalotaxine

Plasma and urine samples collected through 72 h after injection were additionally analyzed using validated liquid chromatography-tandem mass spectrometry assays, described elsewhere [20], to quantify unchanged omacetaxine and the metabolites 4′-DMHHT and cephalotaxine (Fig. 1).

Plasma samples were prepared using protein precipitation with acetonitrile-methanol (80:20, *v/v*), and urine samples were prepared using solid-phase extraction. Both plasma and urine samples were separated on an XBridge BEH Phenyl column (50 × 2.1 mm internal diameter, particle size 5 μm; Waters, Etten-Leur, The Netherlands). Detection was performed with a QTrap 5500 MS/MS (Sciex, Thornhill, ON, Canada) equipped with a turbo ion spray interface, operating in positive mode and configured in multiple-reaction monitoring. The validated calibration range of omacetaxine, 4′-DMHHT, and cephalotaxine in plasma was 0.1 to 100 ng/mL; in urine it was 0.1 to 50 ng/mL. Thus, the LLOQ was defined as 0.10 ng/mL for omacetaxine and its metabolites. Quality control samples were prepared and analyzed together with the study samples. Acceptance criteria for bioanalytical data during routine sample analysis, as described in the FDA and European Medicines Agency guidelines, were applied [21, 22].

### Pharmacokinetic analysis

The pharmacokinetic analysis set included those patients for whom at least one pharmacokinetic parameter could be calculated postbaseline. Pharmacokinetic parameters were estimated using noncompartmental analysis with Phoenix™ WinNonlin® (version 6.3, Pharsight Corporation, Mountain View, CA, USA). The following pharmacokinetic parameters were estimated for omacetaxine, 4′-DMHHT, cephalotaxine, and TRA when possible: maximum observed plasma concentration (C_max_), time to C_max_ (t_max_), terminal elimination half-life (t_1/2_), and area under the plasma concentration-time curve (AUC). For omacetaxine, the apparent plasma clearance (CL/F), apparent volume of distribution (V_z_/F), renal clearance (CL_R_), and metabolite-to-parent AUC ratios were also determined.

### Safety assessments

The safety of omacetaxine was assessed by grading AEs according to National Cancer Institute Common Terminology Criteria for AEs (NCI CTCAE) version 4.0, clinical laboratory test results (hematology, serum chemistry, and/or urinalysis), vital signs and body weight measurements, electrocardiography and physical examination results, and concomitant medication usage.

### Statistical analysis

No formal statistical analysis was applied in this study; descriptive statistics were used when appropriate.

## Results

### Patients

Six patients with advanced solid tumors (three with colorectal, two with lung, and one with ovarian) were enrolled and completed period A of the study (Table 1). One patient with colorectal cancer was withdrawn from the study at the end of period A because of a grade 2 serious AE of constipation (not related to study treatment). Five patients continued into period B. None of the patients completed six cycles of omacetaxine; three patients completed one cycle, and two completed two cycles. Reasons for early discontinuation were lack of efficacy (three patients), patient decision (one patient), and general deterioration (one patient).

**Table 1:** Baseline patient characteristics (*N* = 6)

Characteristic	Value
Median age, years (range)	57.5 (43.0–69.0)
Sex, n (%)	
Male	1 (17)
Female	5 (83)
Race, n (%)	
White	6 (100)
Ethnicity, n (%)	
Not Hispanic or Latino	6 (100)
Median weight, kg (range)	85.1 (70.6–90.2)
Median height, cm (range)	171 (165–184)
Median body surface area, m^2^ (range)	2.0 (1.8–2.1)
Median time since cancer diagnosis, months (range)	26.6 (18.1–42.0)
Primary cancer types, n (%)	
Lung	2 (33)
Colorectal	3 (50)
Ovarian	1 (17)
Received prior cancer drug therapy, n (%)	6 (100)
ECOG performance status, n (%)	
1	4 (67)
2	2 (33)

*ECOG,* Eastern Cooperative Oncology Group

All six patients were white; five were female and one was male, with a mean age of 56.7 years (range 43–69), a mean weight of 81.9 kg (range 70.6–90.2), a mean height of 174 cm (range 170–184), and a mean body surface area of 2.0 m^2^ (range 1.8–2.1). All patients completed period A and were therefore evaluable for safety and pharmacokinetic analysis.

### Pharmacokinetics

**Fig. 2:**
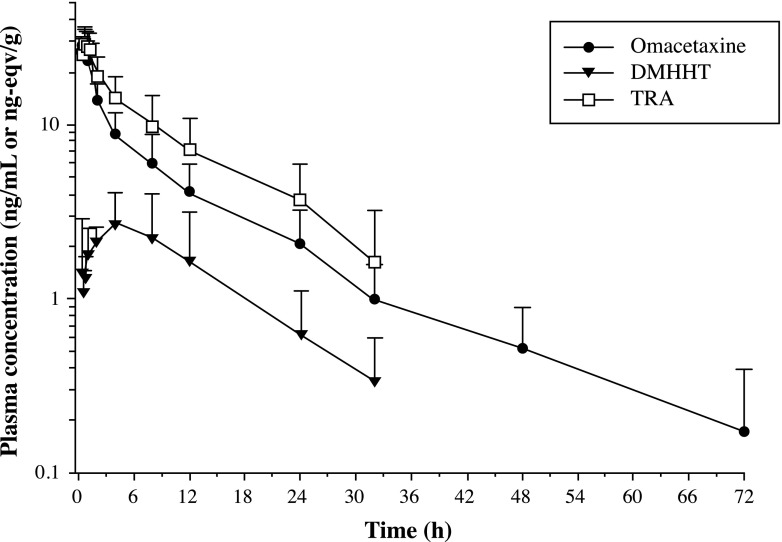
Mean (+ standard deviation) log-linear plasma concentration-time curves of total radioactivity (TRA), omacetaxine and 4′-DMHHT (DMHHT) in plasma (*N* = 6)

Plasma concentration-time curves of TRA, omacetaxine, and 4′-DMHHT through 72 h after the administration of ^14^C-omacetaxine are presented in Fig. 2. A summary of pharmacokinetic parameters is displayed in Table 2.

**Table 2:** Plasma pharmacokinetic parameters for total radioactivity, omacetaxine, and its metabolite 4′-DMHHT following a single subcutaneous dose of 1.25 mg/m^2^ (95 μCi) ^14^C-omacetaxine (*N* = 6)

	C_max_ (ng/mL)^a^	t_max_ (h)	AUC_0-t_ (ng.h/mL)^b^	AUC_0-inf._ (ng.h/mL)^b^	t_1/2_ (h)	V_z_/F (L)	CL/F (L/h)	M:P ratio (AUC_0-inf_)
TRA								
Mean	30.9	0.6^c^	262	292	11.3	ND	ND	ND
SD	6.1	0.3–1.0^c^	127	134	2.5	ND	ND	ND
Omacetaxine								
Mean	28.6	0.48^c^	180	188	14.6	268	14.4	ND
SD	6.5	0.3–0.5^c^	75.4	79.9	5.5	59.3	6.2	ND
4′-DMHHT								
Mean	3.5	3.98^c^	45.3	49.6	10.7	ND	ND	0.27
SD	1.3	0.2–8.1^c^	33.0	34.1	2.9	ND	ND	0.14

*AUC*_*0-t*_ area under the plasma concentration-time curve from time zero to time of the last quantifiable concentration,, *AUC*_*0-inf*_ area under the plasma concentration-time curve from time zero to infinity, *CL/F* total plasma clearance, *C*_*max*_ maximum observed plasma concentration, *M:P* metabolite-parent, *NA* not applicable, *ND* not determined, *SD* standard deviation, *t*_*1/2*_ terminal elimination half-life, *t*_*max*_ time to maximum observed plasma concentration, *TRA* total radioactivity, *V*_*z*_ */F* apparent volume of distribution

^a^Units are ng-eqv/g for TRA

^b^Units are ng-eqv∙h/g for TRA

^c^Median/range values presented

After administration of a single dose of ^14^C-omacetaxine, t_max_ of TRA and omacetaxine were recorded at approximately 0.5 h. After reaching peak levels, the mean plasma concentrations of omacetaxine declined in a biphasic manner that was characterized by an initial rapid phase followed by a slower terminal phase with a mean half-life of 14.6 h (Fig. 2). Observed plasma concentrations for 4′-DMHHT were substantially lower than for omacetaxine. After reaching peak levels, 4′-DMHHT concentrations declined in a monophasic manner.

The metabolite cephalotaxine was quantifiable in only one plasma sample from a single patient at 15 min after administration of ^14^C-omacetaxine.

**Fig. 3:**
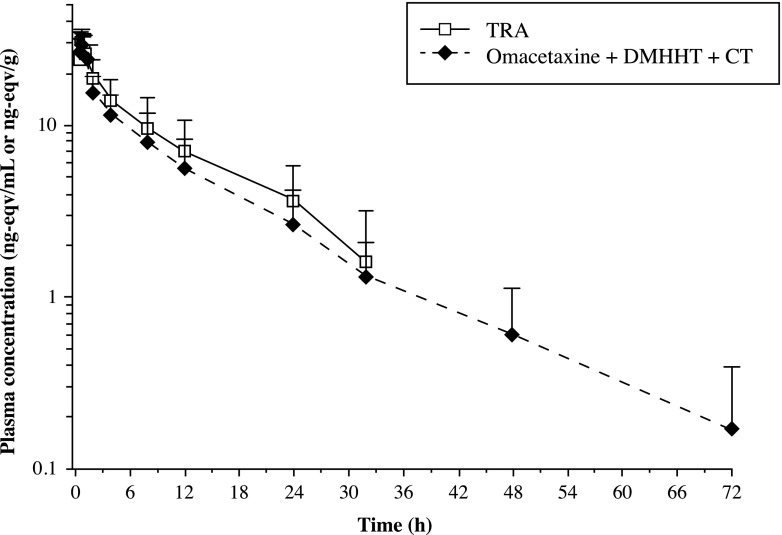
Mean (+standard deviation) log-linear plasma concentration-time curves of total radioactivity (TRA), omacetaxine, 4′-DMHHT (DMHHT), and cephalotaxine (CT) in plasma (*N* = 6)

During the first hour postdose, TRA concentrations were comparable to those for omacetaxine (Fig. 2). Subsequently, the TRA and omacetaxine concentration-time profiles began to diverge but declined in parallel. In a comparison of the concentrations of omacetaxine, 4′-DMHHT, and cephalotaxine to TRA concentrations within the first hour postdose, more than 90 % of the TRA was accounted for by the sum of the three compounds (Fig. 3). At subsequent time points through 32 h postdose, these three compounds comprised approximately 70 to 80 % of the TRA.

At 0.5 h and 8 h postdose, the plasma-to-whole blood concentration ratios of TRA were 1.40 and 1.39, respectively. The ratios were not calculable for samples collected at 72 or 168 h postdose, because TRA concentrations in both matrices at those time points were below the limit of quantitation.

### Excretion balance

**Fig. 4:**
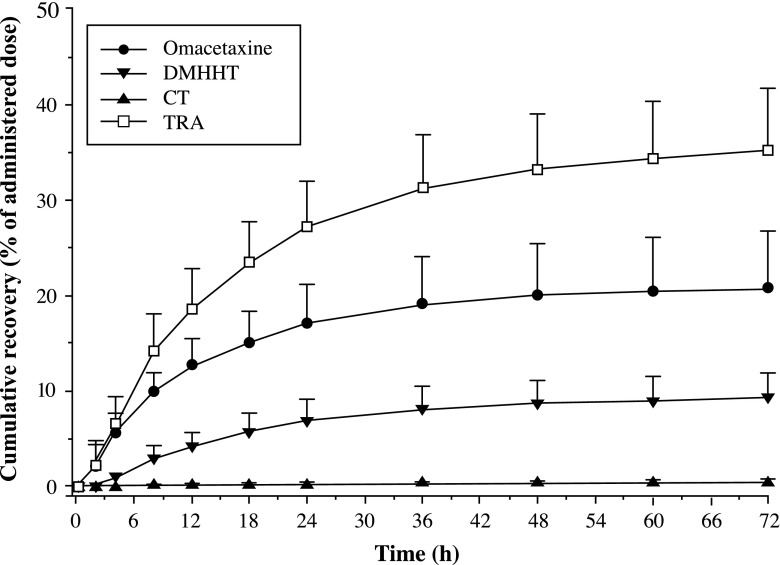
Mean (+standard deviation) cumulative urinary excretion of total radioactivity (TRA), omacetaxine, and metabolites 4′-DMHHT (DMHHT) and cephalotaxine (CT) up to 72 h after a subcutaneous injection (1.25 mg/m^2^) of ^14^C-omacetaxine (*N* = 6)

**Fig. 5:**
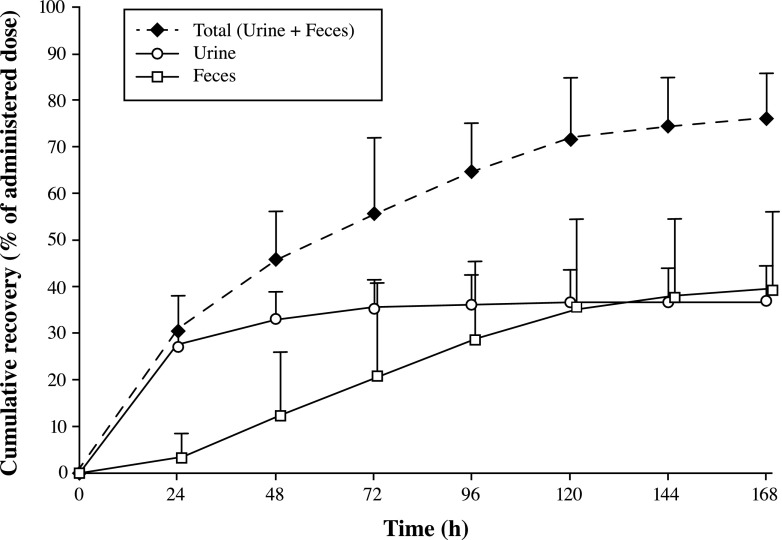
Mean (+standard deviation) cumulative recovery of total radioactivity in urine, feces, and total excreta during 168 h after a subcutaneous injection (1.25 mg/m^2^) of ^14^C-omacetaxine (*N* = 6)

Table 3 and Figs. 4 and 5 summarize the mean cumulative excretion in urine of TRA, unchanged omacetaxine, 4′-DMHHT, and cephalotaxine during the first 72 h and the mean cumulative urinary, fecal, and total recovery of TRA during 168 h after administration of a single subcutaneous dose of ^14^C-omacetaxine.

**Table 3:** Recovery of omacetaxine and metabolites in urine after 72 h, and of total radioactivity in excreta after 168 h and after the total recovery period (range, 144–312 h postdose) (*N* = 6)

	Mean [Min, Max], %	SD, %
Cumulative % recovered in urine up to 72 h		
Omacetaxine	20.7 [15.3, 32.1]	6.0
4′-DMHHT	9.2 [5.5, 13.1]	2.6
Cephalotaxine	0.4 [0.2, 0.6]	0.2
Total	30.3 [22.8, 42.9]	6.7
TRA	35.2 [25.9, 42.9]	6.4
Cumulative % TRA recovered up to 168 h		
Urine	36.9 [26.8, 45.8]	7.6
Feces	39.3 [20.8, 57.2]	16.6
Total	76.2 [66.3, 89.3]	9.7
Cumulative % TRA recovery after total collection period		
Urine	37.0 [26.8, 45.8]	7.7
Feces	43.5 [24.8, 57.5]	13.7
Total	80.5 [68.7, 89.9]	8.7

*Max* maximum, *Min* minimum, *SD* standard deviation, *TRA* total radioactivity

Urinary recovery of omacetaxine and 4′-DMHHT was 20.7 and 9.2 %, respectively, of the administrated dose, and recovery of cephalotaxine was low (0.4 %) in the first 72 h (Table 3). Excretion was moderately slow for all three compounds, with the bulk of urinary recovery occurring within 36 h postdose (Fig. 4).

Over the 168 h of sample collection, the mean cumulative urinary recovery of TRA was 36.9 % of the administered radiolabeled dose. Most of this TRA (35.2 %) was excreted in the first 72 h postdose. The recovery of TRA in 72 h was slightly higher than the sum of the recoveries of omacetaxine and its two metabolites over this same period (35.2 % versus 30.3 %).

Substantial fecal excretion of TRA was also observed in these patients. After 168 h, the mean total recovery of TRA in feces was 39.3 % of the administered radiolabeled dose. In contrast to urinary recovery, in which the majority of the TRA was excreted during the first day, fecal excretion occurred much more slowly (Fig. 5).

The mean total recovery in excreta at 168 h after administration represented 76.2 % of the administered radiolabeled dose; however, small amounts of radioactivity were still present in the last scheduled collection of excreta of some patients. Therefore, urine and/or feces collections continued beyond 168 h for all but one patient, whose collections ended at 144 h because less than 1 % of the administered radioactivity had been excreted in both matrices on that day. The mean total recovery in excreta after the total collection period (range, 144–312 h postdose for individual patients) was 80.5 % of the administered dose.

### Safety

Each of the six enrolled patients experienced at least one AE (100 %). A summary of treatment-emergent AEs is presented in Table 4; the most frequently reported were fatigue (83 %), anemia (50 %), nausea (50 %), and back pain (50 %). The majority of AEs were grade 1 or 2 in severity (NCI CTCAE v4.0). The most common grade 3 AE was anemia (33 %). Other grade 3 AEs experienced by one patient each were abdominal pain, fatigue, hypophosphatemia, and dyspnea. Thrombocytopenia was the most common grade 4 AE; grade 4 neutropenia was experienced by one patient. Three patients experienced a total of 4 serious AEs that included grade 2 constipation, grade 4 thrombocytopenia, grade 3 anemia, and grade 3 dyspnea.

**Table 4:** Treatment-emergent adverse events (*N* = 6)

System organ class: MedDRA 16.0 preferred term	Patients, n (%)			
	Grade 1 AE	Grade 2 AE	Grade 3 AE	Grade 4 AE
Blood and lymphatic system disorders	0	0	1 (17)	2 (33)
Anemia	0	1 (17)	2 (33)	0
Neutropenia	0	0	0	1 (17)
Thrombocytopenia	0	0	0	2 (33)
Cardiac disorders	0	1 (17)	0	0
Atrial fibrillation	0	1 (17)	0	0
Gastrointestinal disorders	2 (33)	3 (50)	1 (17)	0
Abdominal pain	1 (17)	0	1 (17)	0
Abdominal pain upper	1 (17)	0	0	0
Constipation	0	2 (33)	0	0
Diarrhea	1 (17)	1 (17)	0	0
Nausea	3 (50)	0	0	0
Vomiting	1 (17)	0	0	0
General disorders and administration site conditions	0	4 (67)	1 (17)	0
Fatigue	0	4 (67)	1 (17)	0
Influenza-like illness	1 (17)	0	0	0
Injection site rash	1 (17)	0	0	0
Pyrexia	1 (17)	0	0	0
Infections and infestations	0	1 (17)	0	0
Cystitis	0	1 (17)	0	0
Investigations	0	0	0	1 (17)
Blood bilirubin increased	0	1 (17)	0	0
Neutrophil count decreased	0	0	0	1 (17)
Platelet count decreased	0	0	1 (17)	0
White blood cell count decrease	0	0	1 (17)	0
Metabolism and nutrition disorders	1 (17)	0	1 (17)	0
Hypocalcaemia	1 (17)	0	0	0
Hypokalemia	1 (17)	0	0	0
Hypophosphatemia	0	0	1 (17)	0
Musculoskeletal and connective tissue disorders	3 (50)	2 (33)	0	0
Back pain	1 (17)	2 (33)	0	0
Myalgia	2 (33)	0	0	0
Pain in extremity	1 (17)	0	0	0
Psychiatric disorders	0	1 (17)	0	0
Anxiety	0	1 (17)	0	0
Reproductive system and breast disorders	1 (17)	0	0	0
Pelvic pain	1 (17)	0	0	0
Respiratory, thoracic and mediastinal disorders	0	2 (33)	1 (17)	0
Cough	0	1 (17)	0	0
Dyspnea	0	1 (17)	1 (17)	0
Skin and subcutaneous tissue disorders	1 (17)	0	0	0
Erythema	1 (17)	0	0	0
Vascular disorders	1 (17)	0	0	0
Flushing	1 (17)	0	0	0

*AE* adverse event, *MedDRA* Medical Dictionary for Regulatory Activities

If a patient reported an AE more than once, the greatest severity is presented for that AE. Patients were counted only once in each preferred term category and only once in each system organ class category, at the greatest severity for each

No deaths occurred in this study. One patient with colorectal cancer experienced grade 2 constipation on day 2 of the study that necessitated hospitalization and was resolved on day 7. This patient withdrew from the study on day 8 because of a second serious AE of constipation. The lowest recovery of TRA was obtained in this patient (68.7 % versus 70.6 to 89.9 % for the other five patients), but the pattern of excretion between urine and feces was similar. This event was reported as recovered/resolved with sequelae on day 15, but the patient continued to experience further grade 1 constipation. None of these events were considered to be related to omacetaxine.

Assessment of clinical laboratory parameters revealed grade 1 serum chemistry changes for alkaline phosphatase, calcium, glucose, potassium, and aspartate aminotransferase/alanine aminotransferase. One patient experienced a grade 3 serum glucose increase that was related to an underlying medical condition and unrelated to study treatment. Laboratory hematologic findings included grade 4 neutropenia, grade 3/4 leucopenia, and grade 3/4 thrombocytopenia in three patients each and grade 3 lymphopenia in two patients. All grade 3/4 hematologic findings were not serious and were considered related to study drug. One patient experienced grade 3 anemia and grade 4 thrombocytopenia reported as a serious AE. The hematologic findings were consistent with the known side-effect profile of omacetaxine and with findings in a patient population heavily pretreated with chemotherapy.

No significant changes in electrocardiography were attributed to omacetaxine, and no QTc prolongation was evident. One patient, with a history of atrial fibrillation, experienced nonserious grade 2 atrial fibrillation on day 1 was treated with an increased dose of digoxin. The atrial fibrillation was considered not related to the study drug and resolved on day 3. Changes in performance status were based on progression of disease, and physical findings were otherwise those expected for this patient population.

## Discussion and conclusions

This study investigated the disposition and elimination of ^14^C-omacetaxine in adult patients with solid tumors following a single subcutaneous dose. After administration, the observed pharmacokinetic profiles of omacetaxine and 4′-DMHHT were consistent with results from a previous study in patients with cancer [17]. The high apparent volume of distribution suggests that omacetaxine is distributed extensively into the tissues; this is consistent with previous reports in which the drug was administered either as a subcutaneous injection or an intravenous infusion [17, 23]. The longer half-life and larger systemic exposure (AUC) to omacetaxine and 4′-DMHHT observed in this study compared with the previous study are attributed to the longer sampling period used (72 versus 12 h).

The summed concentrations of omacetaxine and its metabolites accounted for more than 90 % of the TRA concentrations through the first hour after administration. Subsequently, the concentration profiles diverged, and, at 32 h postdose, the three compounds accounted for approximately 70 % of the TRA. These results suggest that other ^14^C-omacetaxine-derived materials are present in plasma at later hours postdose.

The ratio of plasma to blood TRA concentrations were close to unity over the two time points assessed, suggesting that there was no preferential association of ^14^C-omacetaxine-derived radioactivity with cellular components of the blood.

The mean total recovery of TRA in excreta during the collection period of up to 312 h was 80.5 %. The large apparent volume of distribution and associated potential tissue binding might explain why higher excretion recovery was not achieved. This is in line with the radioactivity recovery in previous mass balance studies of chemotherapy agents [24]. Samples were collected until excretion declined to less than 1 % of the administered dose over 24 h. A higher recovery could have been obtained if the collection time had been extended, but the added value of additional excretion data was considered limited.

Half of the radioactivity was excreted in urine and half in feces, which suggests that both hepatic and renal processes contribute to the elimination of omacetaxine. The elimination of omacetaxine, 4′-DMHHT, and cephalotaxine in urine was moderately slow, with the majority excreted within 36 h postdose. The excretion data reported here for omacetaxine, 4′-DMHHT, and cephalotaxine within the first 24 h after administration correspond to results reported previously by Nemunaitis et al., who found urinary excretion of omacetaxine, 4′-DMHHT, and cephalotaxine to be 12 to 15 %, 4 to 5 %, and 0.07 %, respectively [17]. The recovery of TRA in 72 h was slightly higher than the sum of the recoveries of omacetaxine and its two metabolites over this same period (35.2 % versus 30.3 %), suggesting that other ^14^C-omacetaxine-derived materials may be present. Fecal excretion occurred much more slowly than did excretion in urine and showed larger deviations, likely related to variation in the amount of fecal excretion per patient. Metabolites of omacetaxine have not been extensively characterized in preclinical studies, but the amount of radioactivity and unchanged omacetaxine in urine and feces suggests that the processes involved in the elimination of omacetaxine are similar in humans, dogs, and mice [15, 23, 25].

Treatment with omacetaxine in this heavily pretreated patient population with advanced solid tumors was tolerated, with an AE profile consistent with that known for omacetaxine and those related to the underlying malignancies of these patients. Fatigue, anemia, thrombocytopenia, neutropenia, diarrhea, and nausea were the most common treatment-related AEs reported and were expected. There were no clinically meaningful changes from baseline in serum chemistries, and the hematologic parameter changes were consistent with the known side-effect profile of omacetaxine. Therefore, no new safety signals were observed during the course of this study.

In conclusion, results from this study suggest that both renal and hepatic processes contribute to the elimination of ^14^C-omacetaxine-derived radioactivity in patients with solid tumors. In addition to omacetaxine and its known metabolites, other ^14^C-omacetaxine-derived materials appear to be present in both plasma and urine. Omacetaxine was adequately tolerated by these patients with end-stage cancer, and no new safety signals were observed.
